# Hyperparameter Tuning of Artificial Neural Networks for Well Production Estimation Considering the Uncertainty in Initialized Parameters

**DOI:** 10.1021/acsomega.2c00498

**Published:** 2022-06-14

**Authors:** Miao Jin, Qinzhuo Liao, Shirish Patil, Abdulazeez Abdulraheem, Dhafer Al-Shehri, Guenther Glatz

**Affiliations:** Department of Petroleum Engineering, King Fahd University of Petroleum & Minerals, 31261 Dhahran, Saudi Arabia

## Abstract

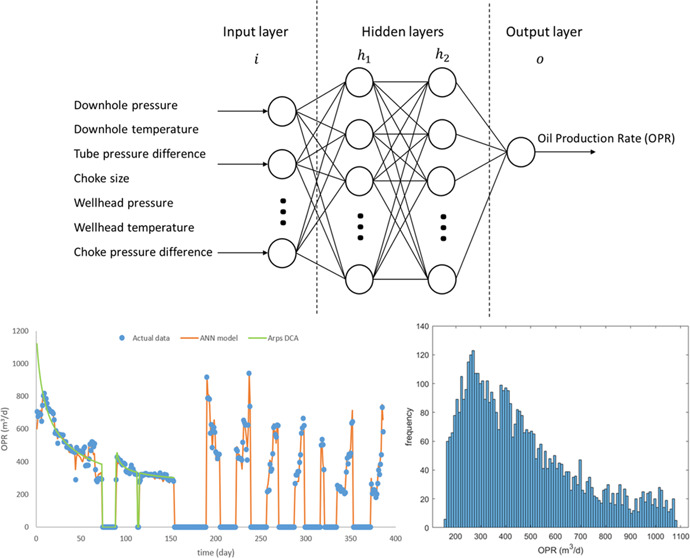

A well production rate is an essential parameter in oil and gas field development. Traditional models have limitations for the well production rate estimation, e.g., numerical simulations are computation-expensive, and empirical models are based on oversimplified assumptions. An artificial neural network (ANN) is an artificial intelligence method commonly used in regression problems. This work aims to apply an ANN model to estimate the oil production rate (OPR), water oil ratio (WOR), and gas oil ratio (GOR). Specifically, data analysis was first performed to select the appropriate well operation parameters for OPR, WOR, and GOR. Different ANN hyperparameters (network, training function, and transfer function) were then evaluated to determine the optimal ANN setting. Transfer function groups were further analyzed to determine the best combination of transfer functions in the hidden layers. In addition, this study adopted the relative root mean square error with the statistical parameters from a stochastic point of view to select the optimal transfer functions. The optimal ANN model’s average relative root mean square error reached 6.8% for OPR, 18.0% for WOR, and 1.98% for GOR, which indicated the effectiveness of the optimized ANN model for well production estimation. Furthermore, comparison with the empirical model and the inputs effect through a Monte Carlo simulation illustrated the strength and limitation of the ANN model.

## 1 Introduction

In reservoir evaluation, the well production rate is one of the critical parameters that helps petroleum engineers to make decisions in exploration and operation. Three conventional approaches were applied for the well production forecast: analytical methods, numerical simulations, and empirical models. Analytical approaches, based on the diffusivity equation for steady-state flow, adopt certain assumptions to simplify the complicated reservoir models to get the solutions and are widely used in the prediction of well flow rates. However, these solutions may not be able to fit the frequent manual operations and state changes in the subsurface multiphase flow.^1^ Numerical models can well describe the reservoirs’ heterogeneity but are usually time-consuming and require various types of data, e.g., logging, porosity, and permeability.

Another way is using empirical equations to predict the well production rate. Decline curve analysis (DCA) is one of the most widely used empirical methods. DCA was first put forward by Arps,^2^ who summarized exponential, harmonic, and hyperbolic decline curves, and developed by subsequent researchers.^3−9^ However, DCA may not accurately estimate the actual well production because of its assumption simplification for well operation. Machine learning (ML) is the subarea of artificial intelligence (AI), which was introduced in the 1950s as “the study field of computer’s ability to learn things without being explicitly programmed”. In brief, it splits the dataset into three parts: training, validation, and testing to build models that serve the purpose.^10^ ML comprises a vast number of models and algorithms. Some of them have become popular in well production forecast in recent decades. For example, Surguchev^11^ used an artificial neural network (ANN) trained with backpropagation and scaled conjugate gradient to evaluate the improved oil recovery (IOR) methods (water flood, air injection, etc.). They chose 12 reservoir parameters (porosity, permeability, viscosity, etc.) as input to evaluate the IOR methods. Cao et al.^12^ utilized ANN to forecast both existing and new well production. They compared the results with DCA models and found that the ANN model has a much better performance. Jia and Zhang^13^ compared ANN with classic DCA models based on different historical data ranges. Vyas et al.^14^ used random forest, supported vector machine (SVM), and multivariate adaptive regression splines (MARS) to predict the early-time decline rate. Li et al.^15^ combined the ANN model with DCA to predict the well production. They use a logistic growth model (a DCA) to fit the well production data from different formation conditions, and the results are three model parameters (carrying capacity, constant, and hyperbolic exponent). Then, the ANN model was constructed with formation conditions as inputs and model parameters as outputs. Finally, logistic growth models could be used to predict well productions when the new formation conditions were obtained for the ANN model. Khan^16^ compared the ANN, adaptive neuro-fuzzy inference system (ANFIS), and SVM for predicting the oil rate in artificial gas lift wells. Li et al.^17^ and Masini et al.^18^ compared various ML models for predicting the downhole pressure, and the process can also be applied to predict well production. Xue et al.^19^ combined the random forest and ensemble Kalman filter to forecast the dynamic transient pressure automatically. Yavari et al.^20^ adopted the ANFIS model to estimate the pressure difference from the end point (toe) to the hell in the lateral section of the horizontal well. ANN models were also applied to the prediction of minimum CO_2_ miscibility pressure.^21,22^ Fan et al. developed an autoregressive integrated moving average-long short-term memory (ARIMA-LSTM) hybrid model to forecast the well production. Yang et al.^23^ constructed a GRU-ANN machine learning hybrid model to predict coalbed methane well production. Liao et al.^24^ applied a hybrid model that used K-means clustering, an unsupervised machine learning method, combined with DCA to predict the well production rate.

However, there are only a few publications that have predicted the water oil ratio (WOR) and gas oil ratio (GOR) along with the oil production rate (OPR). In addition, detailed optimization of the neural network in well production prediction is rarely mentioned in previously reported research. In addition, most existing literature for the neural network optimization is from a deterministic point of view without considering the uncertainty/randomness in the initialization of neural network parameters such as weight and bias. This study aims to optimize the hyperparameters of an artificial neural network in well production estimation, including the types of neural networks, training function, and transfer function. This study applied several evaluation parameters, e.g., average absolute percentage error (AAPE), coefficient of determination (*R*^2^), and relative root mean square error (relative RMSE) and assessed the uncertainty of initialized parameters using some statistical parameters to figure out the optimal ANN models for the production estimation.

## 2 Methodology

### 2.1 ANN Hyperparameters

**Figure 1:**
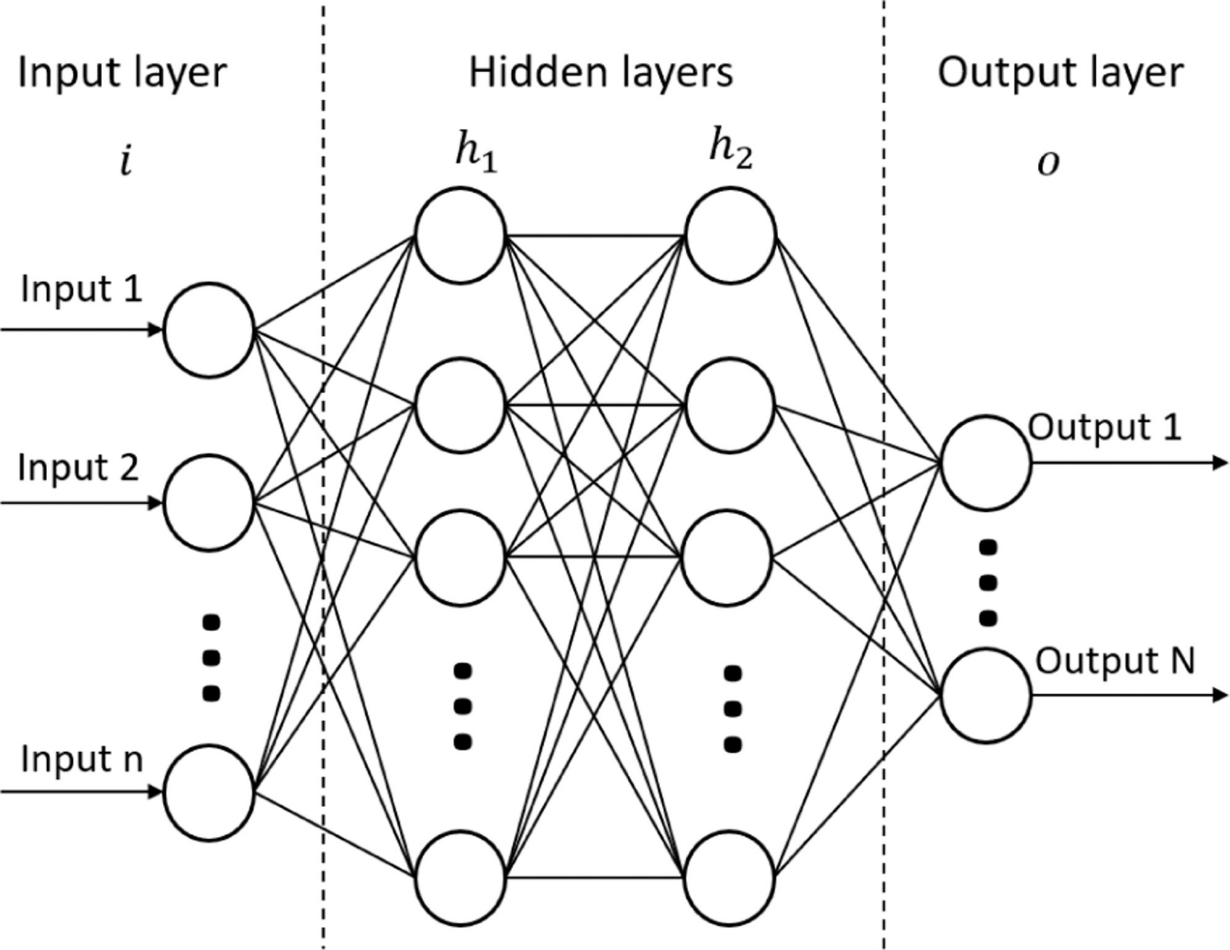
Feedforward neural network, including one input layer (*i*), two hidden layers (*h*_1_,*h*_2_), and one output layer (*o*).

The machine learning method selected in this research is the artificial neural network (ANN), commonly adopted in well production prediction in recent years. ANN is the information-processing algorithm inspired by and mimicking the process of human brain’s biological neural networks. It has the potential to analyze big historical data and is widely applied in regression and classification problems, especially cases that cannot be solved by traditional mathematical models.^25^ Each ANN model consists of sequential layers and connections: the input layer, the (one or more) hidden layers, and the output layer. The layer structure comprises several nodes called neurons (Figure 1). Each neuron contains a built-in function that addresses the received signals, and the neurons in different layers are connected to deliver information.

#### 2.1.1 Neural Network Type

Neural network type is the parameter determining the ANN model structure. This study tested three types of neural networks (feedforward neural network, function fitting neural network, and cascade-forward neural network) in the ANN model construction. Feedforward neural network (“newff” function in MATLAB) is a category of ANN wherein the neurons are connected sequentially and do not form a cycle, which is different from the recurrent neural network. Function fitting neural network (“fitnet” function in MATLAB) is a type of feedforward neural network that is widely used in regression problems. Cascade-forward neural network (“newcf” function in MATLAB) is similar to feedforward neural network except that the input layer has connections to every hidden layer.

**Figure 2:**
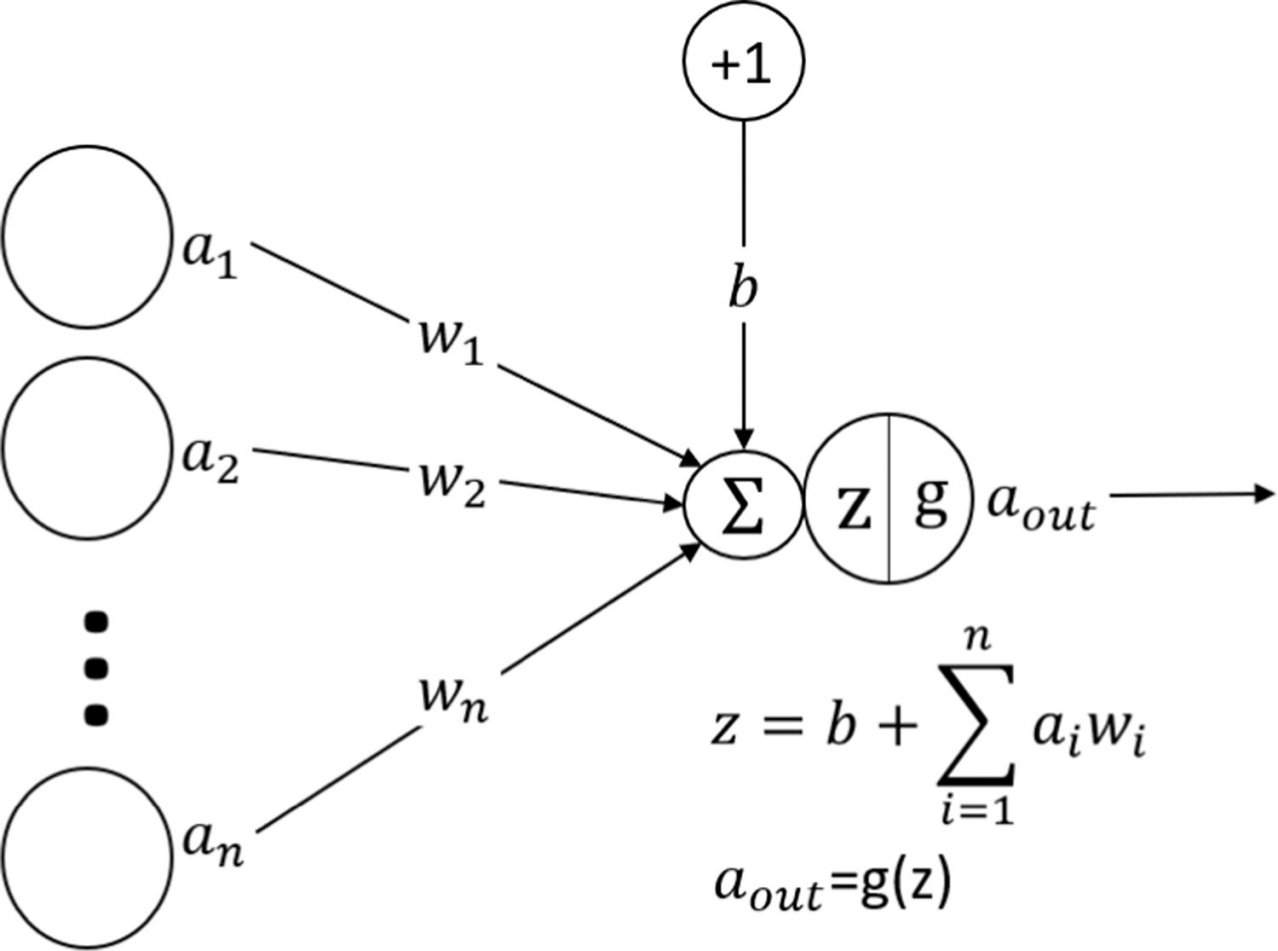
Feedforward process in the neural network.

The basic procedure in the neural network can be described as follows:^26^ the numerical-converted features of observations (inputs) are assigned to the input layer, and each neuron denotes one feature. Then, weight and bias are applied to the inputs when transmitted to the next layer’s neurons. Specifically, each variable in the input layer is multiplied by weight through the connection (each connection has independent weights). All of the transformed variables are then summed in each neuron of the hidden layer with an additional value called bias. After that, the result is converted by the built-in function called the transfer function or activation function in the hidden layer neuron (Figure 2). The process runs simultaneously in neurons of the same layer and is repeated in the following layers until the output layer is reached, where the output is generated.

Here *a*_1_, *a*_2_, and *a_N_* are the inputs, *w*_1_, *w*_2_, and *w_N_* are the weights for the corresponding inputs, *b* is the extra bias value, *z* is the sum of weighted inputs and bias, *g* is the built-in transfer function, and *a*_out_ is the hidden layer neuron output.

#### 2.1.2 Training Function

Weights and biases are updated by backpropagation, an algorithm applying the chain rule and derivative of error function, in the ANN model. Training functions determine the type of backpropagation algorithm. Several training functions were tested to figure out the optimal training function settings in this case study (Table 1).

**Table 1:** Training Function Setting

training function	abbreviation
Levenberg–Marquardt	trainlm
Bayesian regularization	trainbr
resilient backpropagation	trainrp
BFGS quasi-Newton	trainbfg
conjugate gradient with Fletcher–Reeves updates	traincgf
conjugate gradient with Powell–Beale restarts	traincgb
conjugate gradient with Polak–Ribiére updates	traincgp

#### 2.1.3 Transfer Function

Transfer function is the built-in function inside the neuron, which converts the received information from the previous layer. This study analyzes different transfer function groups to obtain the optimal solution for the prediction model. Table 2 lists the transfer functions adopted in this study.

**Table 2:** Transfer Function Setting^a^

transfer function	abbreviation	equation
soft max	softmax	
log-sigmoid	logsig	
triangular basis	tribas	
saturating linear	satlin	
symmetric saturating linear	satlins	
positive linear	poslin	
linear	purelin	*y* = *x*
radial basis	radbas	*y* = e^–*x*2^
normalized radial basis	radbasn	

a Note: For the soft max and normalized radial basis function, the transformation of input will be normalized, where *m* denotes the *m*th neuron in the layer, and *K* is the neuron number of the layer.

### 2.2 Error Functions

The average absolute percentage error (AAPE) and coefficient of determination (*R*^2^) are two metrics widely applied in data analysis. AAPE is the relative error between the target actual value and the prediction result (eq 1) and reflects the accuracy of the data-driven model. However, AAPE is vulnerable to the extreme value, e.g., the estimation result of 1 with the actual value 0.5 will lead to a 100% AAPE. *R*^2^ is the statistical parameter expressed by the ratio of sum of square of the residual to the total variance of the actual value (eq 2). However, *R*^2^ might be misleading when the variance of the actual data is extremely low, e.g., the variance of the actual value is 0.1 and the actual mean is 100, but the variance of prediction is 0.05, which leads to 50% *R*^2^
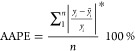
1
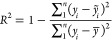
2where *y*_*i*_ is the actual value, *ỹ*_*i*_ is the estimated value, and *y̅* is the mean value of the dataset.

Another common evaluation metric is the root mean square error (RMSE), which measures the residual *s* between the target value and the estimations. However, RMSE may not intuitively reflect the result when compared with different size outputs without normalization, e.g., RMSE comparison of output size 1000 with output size 1. Therefore, the relative RMSE was used to compare the groups’ performance based on the L2 norm (eq 3). It can directly reflect the performance of the ANN model and reduce the influence of the extreme value, which combines the advantage of AAPE and RMSE and avoids their shortcomings
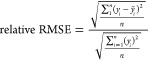
3where *y*_*i*_ is the actual value and *ỹ*_*i*_ is the estimated value.

## 3 Case Study

**Figure 3:**
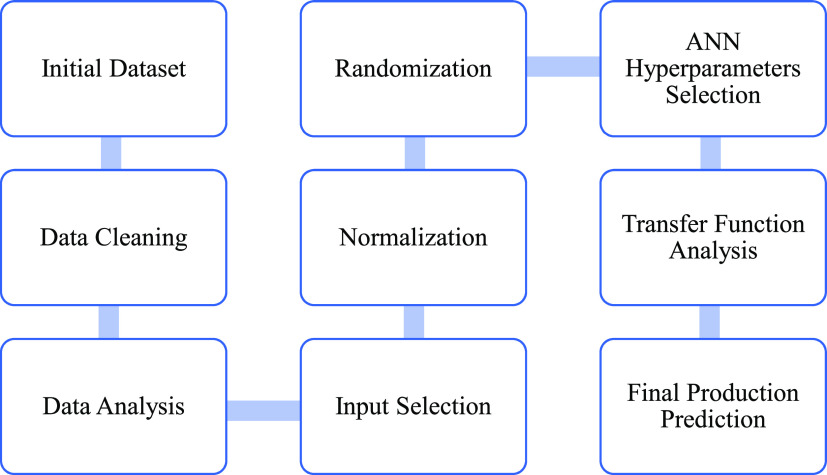
Well production prediction workflow.

This study applies ANN models with two hidden layers to predict OPR, GOR, and WOR. The actual production dataset was initially cleaned and analyzed to select the suitable input parameters. Then, ANN models were applied to predict the target outputs. AAPE and *R*^2^ were adopted to evaluate the ANN model effectiveness under different neural network, training, and single transfer functions. Furthermore, relative RMSE, mean, and standard deviation were applied to determine the best transfer function group for ANN models. Finally, the ANN models with the optimal neural network, training, and transfer functions were selected and predicted the target production parameters. The prediction process is shown in Figure 3.

### 3.1 Data Preparation

The data source in this study comes from the daily production data of a single well belonging to the Volve Field, an offshore field located in Norway.^27^ The data is derived from the multiphase flow period of a production well (oil, gas, water). The shut-in periods are removed since they are manually controlled and unpracticable for WOR and GOR computation. After obtaining the data from the Volve field database, we first cleaned the data to get a valuable dataset. For example, the irrelevant data were removed, e.g., record date and wellbore code. The records containing missing features are removed, e.g., downhole pressure and temperature, and the unreasonable data are modified, e.g., 25 daily on-stream hours. After cleaning, 240 data points were selected for the case study. The dataset contained downhole pressure, downhole temperature, pressure difference of the tube, choke size, wellhead pressure, wellhead temperature, pressure difference of choke, GOR, WOR, and OPR (Table 3). GOR, WOR, and OPR were chosen as outputs to compare with the practical production performance, while other parameters were selected as inputs.

**Table 3:** Well Production Dataset from the Volve Field

abbreviation of the data type	description
DHP	downhole pressure (bar)
DHT	downhole temperature (°C)
DP Tube	tubing pressure difference (bar)
choke size	choke size percentage (%)
WHP	wellhead pressure (bar)
WHT	wellhead temperature (bar)
DP choke size	choke pressure difference (bar)
GOR	gas oil ratio
WOR	water oil ratio
OPR	daily oil production rate (m^3^/d)

### 3.2 Data Analysis

**Figure 4:**
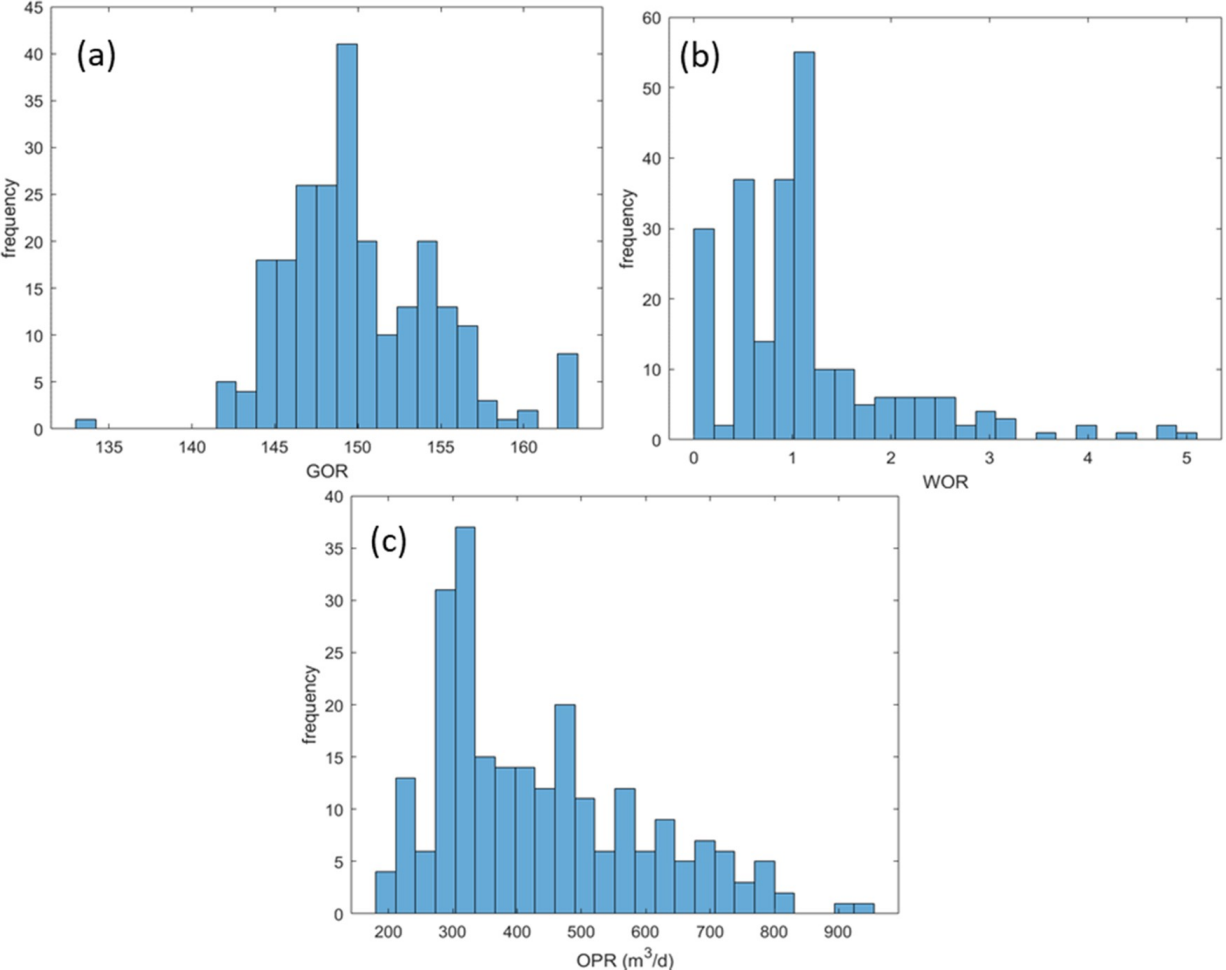
(a) GOR frequency histogram; (b) WOR frequency histogram; and (c) OPR frequency histogram.

The objective of the data analysis is to determine the appropriate inputs for the machine learning models and validate the data cleaning results. Data analysis is composed of the regular analysis and correlation analysis. Normal analysis parameters include minimum and maximum (Table 4). They can be combined with production parameter distribution histograms (Figure 4) to verify the data range and detect the outliers or the missing value of the dataset.

**Table 4:** Relevant Parameters of Data Analysis

	DHP	DHT (°C)	DP tube	choke size	WHP
min	207.22	106.97	154.69	43.934	28.487
max	279.99	108.49	222.06	73.665	95.439
	WHT	DP choke size	GOR	WOR	oil vol
min	43.698	2.3711	133. 91	0.0684	182.64
max	83.421	66.337	163.12	5.0763	940.93

**Figure 5:**
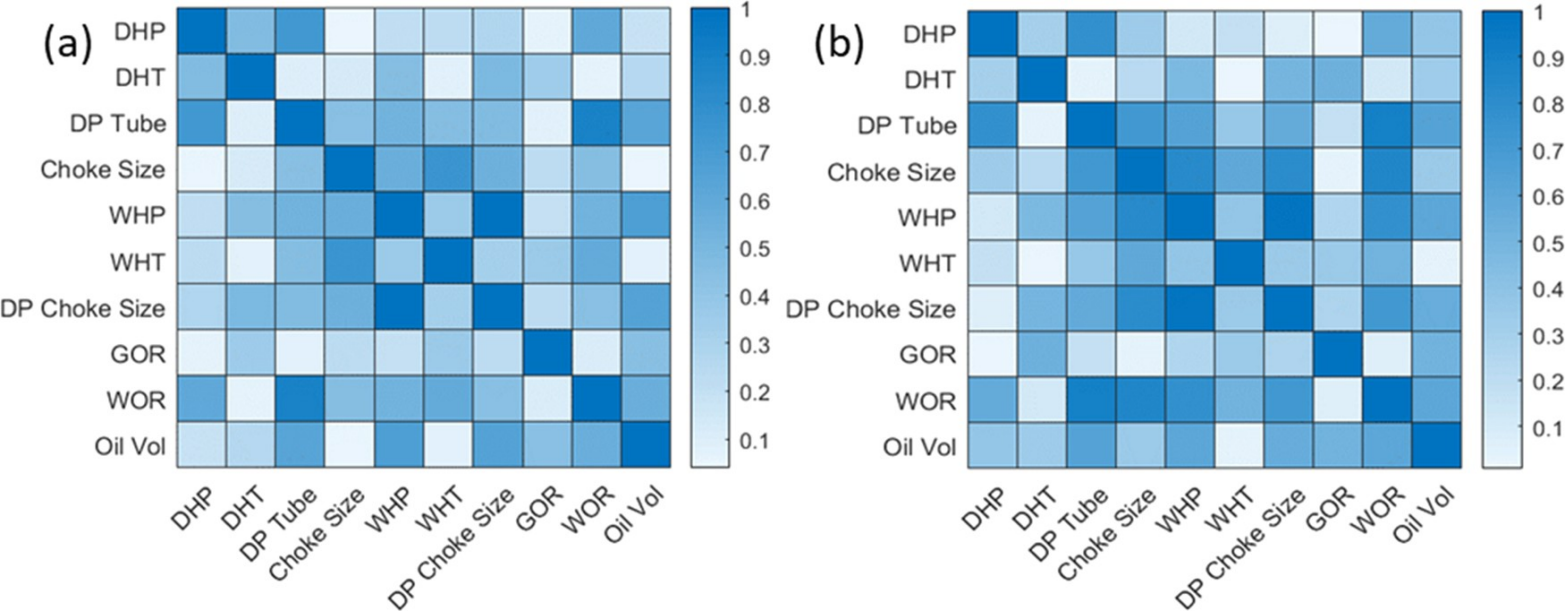
(a) Correlation coefficient heatmap and (b) Spearman correlation coefficient heatmap.

The correlation coefficient and Spearman’s (rank) correlation coefficient are two statistical norms of relationship between two variables. The correlation coefficient describes the linear relationship between two variables. A high absolute value of the correlation coefficient means the connection is strong, while a low absolute value means a weak relationship. Compared with the correlation coefficient, Spearman’s (rank) correlation coefficient represents the monotonic relationship between two variables and could be applied to both linear and nonlinear relationships. For example, in Figure 5a, the absolute correlation coefficient between the choke size and WOR is 0.46. In contrast, in Figure 5b, the spearman absolute correlation coefficient between the choke size and WOR is 0.85. The correlation coefficient means a strong relationship exists between the choke size and WOR, and the association is more likely to be nonlinear.

The inputs for OPR, WOR, and GOR were selected according to the absolute values of the correlation coefficient and Spearman correlation coefficient. The result is shown in Table 5, where “high, medium and low” represent the correlation between the inputs and outputs. The absolute value of the correlation coefficient or Spearman correlation coefficient higher than 0.6 is regarded as the high correlation. In contrast, the absolute values of both correlation coefficient and Spearman correlation coefficient lower than 0.2 are considered low correlation. This study selected the high and medium correlation parameters as inputs for three outputs.

**Table 5:** Input Selection

	OPR	WOR	GOR
downhole pressure	medium	medium	low
downhole temperature	medium	low	medium
tube pressure difference	high	high	low
choke size	medium	high	low
wellhead pressure	medium	medium	medium
wellhead temperature	low	medium	medium
choke pressure difference	medium	high	medium

### 3.3 Model Hyperparameter Selection

After selecting inputs, the ANN model was adopted to estimate the OPR, WOR, and GOR. First, we normalized the inputs to make them have the same range. Then, we randomly chose the data points for training and testing. Two hundred and forty data points were used in our case, and 168 points were selected as the training part, while the remaining were testing data points. The neural network had two hidden layers, and each hidden layer had 20 neurons.

**Figure 6:**
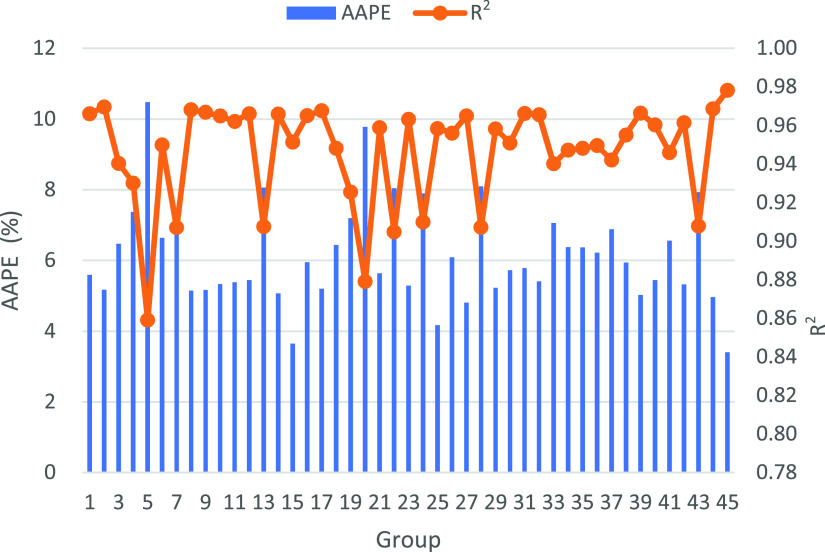
Each group’s *R*^2^ and AAPE for OPR. The best *R*^2^ reaches 0.978 and AAPE reaches 3.5%.

**Figure 7:**
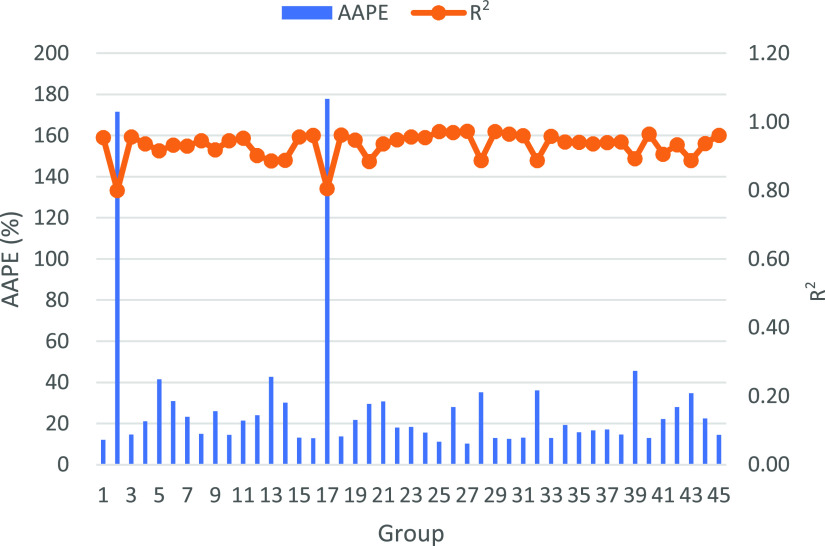
Each group’s *R*^2^ and AAPE for WOR. The best *R*^2^ reaches 0.973 and AAPE reaches 10%.

**Figure 8:**
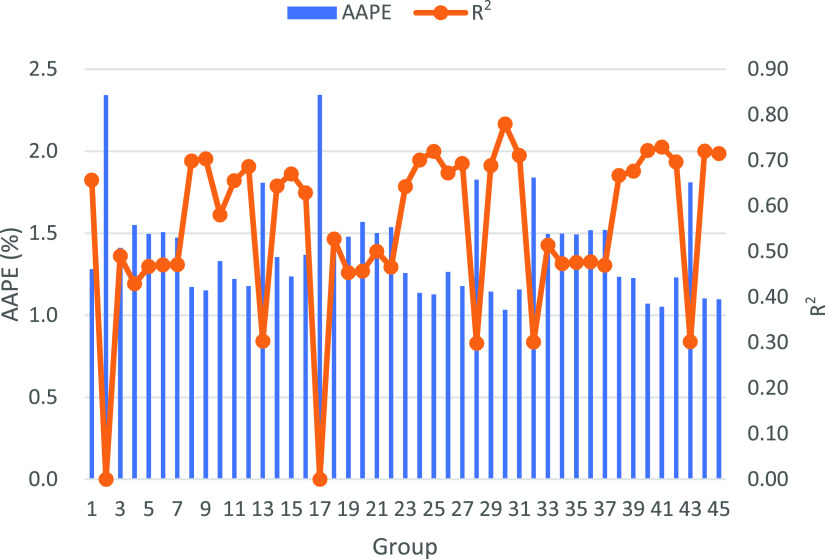
Each group’s *R*^2^ and AAPE for GOR. the best *R*^2^ reaches 0.780 and best AAPE reaches 1.0%.

This study evaluated three types of neural networks (function fitting neural network, feedforward neural network, and cascade-forward neural network) combined with various training and transfer functions (the hidden layers in this section have the same transfer function). There were 45 hyperparameter groups for each output (Appendix A). First, the network and transfer functions were fixed, and several training functions or backpropagation algorithms were attempted to select the best one. Then, different transfer functions or activity functions were tested based on the suitable training function. After that, the network function was changed and the same process was repeated. We ran the ANN model and recorded the case with each hyperparameter group’s lowest average absolute percentage error (AAPE) and highest coefficient of determination (*R*^2^) (Figures 6–8).

The results indicated a high accuracy of the ANN model estimation. The AAPE of the best ANN hyperparameters group for OPR, WOR, and GOR reach 5, 10, and 1%, while the highest *R*^2^’s reach 0.985, 0.986, and 0.88. Moreover, the different results for each output revealed the significance of the selection of ANN hyperparameters. In addition, for the same output estimation, e.g., production rate, the groups with lower AAPE usually have higher *R*^2^ values, which denotes the consistency of the evaluation parameters. However, AAPE and *R*^2^ may show different results when compared with different outputs estimation. For example, the prediction for GOR has a lower AAPE than WOR, but the *R*^2^ for GOR shows a lower value than WOR. The difference could be explained from mathematical and physics aspects. According to the definition of AAPE (eq 1), the relative error of GOR estimation was much lower than WOR. However, due to the small relative range of the GOR value compared with WOR, the ratio of the sum of square residual to the total variance of GOR is bigger than WOR (eq 2), and thus the *R*^2^ of GOR is smaller than that of WOR. Furthermore, the relatively small variability of GOR indicates that the majority of gas in place is stored as the solution gas.

Based on the prediction evaluation, we selected the best prediction group for OPR, WOR, and GOR (Table 6). It should be noted that the transfer functions in two hidden layers were kept consistent in this section. In the latter section, we further explored the influence of various transfer function groups for the prediction based on the current optimal network and training function.

**Table 6:** Optimal Hyperparameter Group for each Output^a^

hyperparameter	OPR	WOR	GOR
network function	newcf	newff	newff
training function	trainbr	trainlm	trainlm
transfer function	radbasn	poslin	radbasn

a Note: Specific names of hyperparameters are included in Appendix A.

### 3.4 Transfer Function Analysis

In the previous section, we set the two hidden layers with the same transfer function. This section performed sensitivity analysis on transfer functions, and our objective is to evaluate the influence of different transfer functions on the prediction results and seek to choose the combination of transfer functions with the lowest error in two hidden layers.

**Figure 9:**
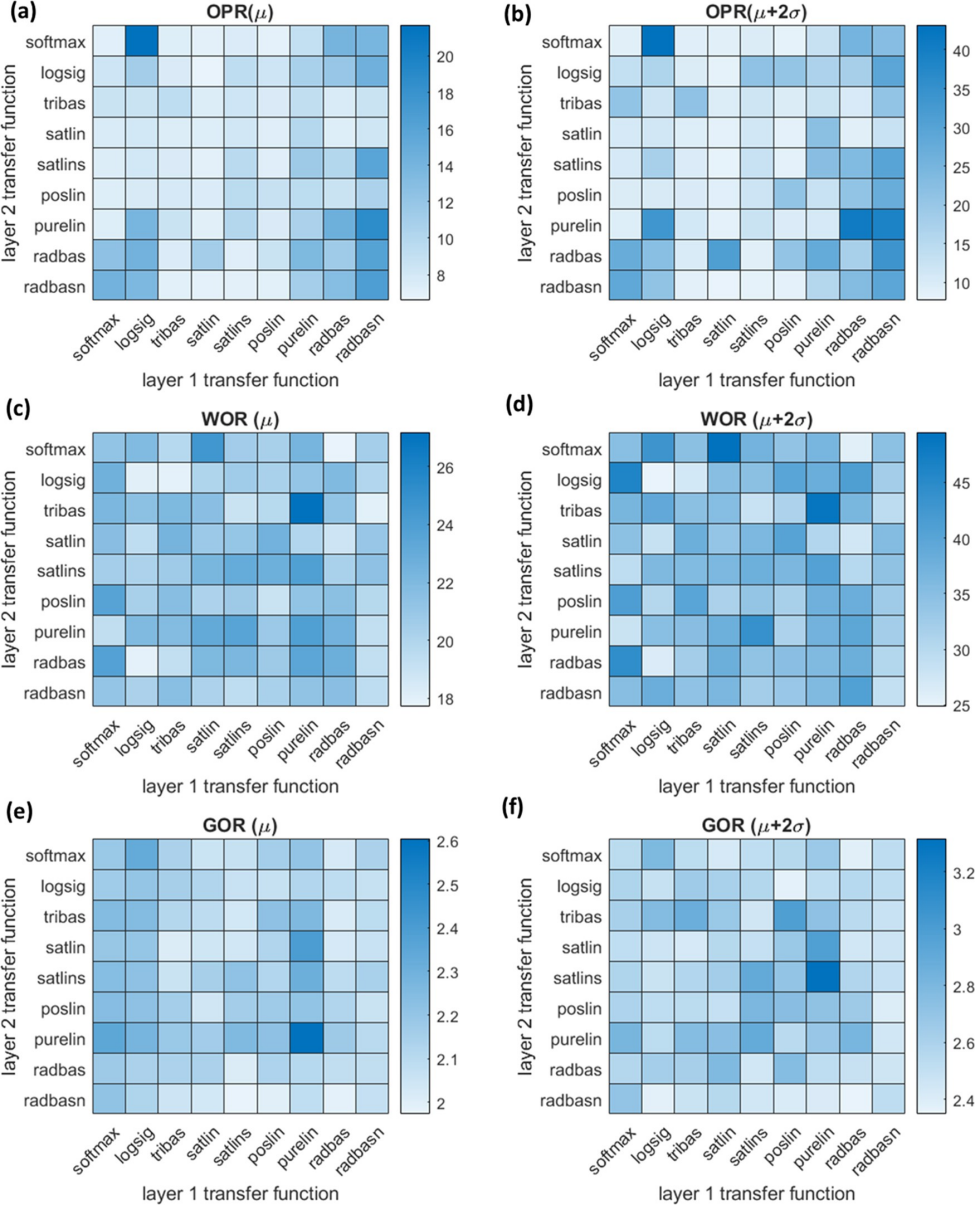
Heatmaps of evaluation for relative RMSE of ANN models. (a) μ for OPR; (b) μ + 2σ for OPR; (c) μ for WOR; (d) μ + 2σ for WOR; (e) μ for GOR; and (f) μ + 2σ for GOR.

The transfer function is the only variable in analysis based on the given suitable network function and training function from the above steps. In our case, we attempted nine transfer functions, and there were 81 groups in total. We ran each group tens of times to reduce the influence of randomization inside the neural network. In this study, mean (μ) and mean plus two standard deviations (μ + 2σ) of the relative RMSE, inspired by the Gaussian distribution, are adopted as the criteria for ANN models’ performance evaluation. The result of two metrics for 81 transfer function groups are plotted in Figure 9. Based on the heatmap, the optimal hyperparameter group for each output was chosen, and their transfer functions are listed in Table 7.

**Table 7:** Optimal Transfer Function Group for Each Output^a^

transfer	OPR	WOR	GOR
layer 1	satlin	logsig	radbas
layer 2	radbasn	logsig	radbasn

a Note: specific names of transfer functions are included in Appendix A.

Several interesting phenomena could be observed from the heatmap. First, the low relative RMSE values showed the effectiveness of ANN models for output estimation. In addition, the similarity between mean and mean plus two standard deviations heatmaps verified the usefulness of the evaluation metrics. Finally, the different results for the same transfer function group in different outputs certified no universal setting for the optimal ANN model. After considering the mean and mean plus two standard deviations relative RMSE, optimal transfer function groups were selected for the ANN model construction (Table 7).

### 3.5 Results

**Figure 10:**
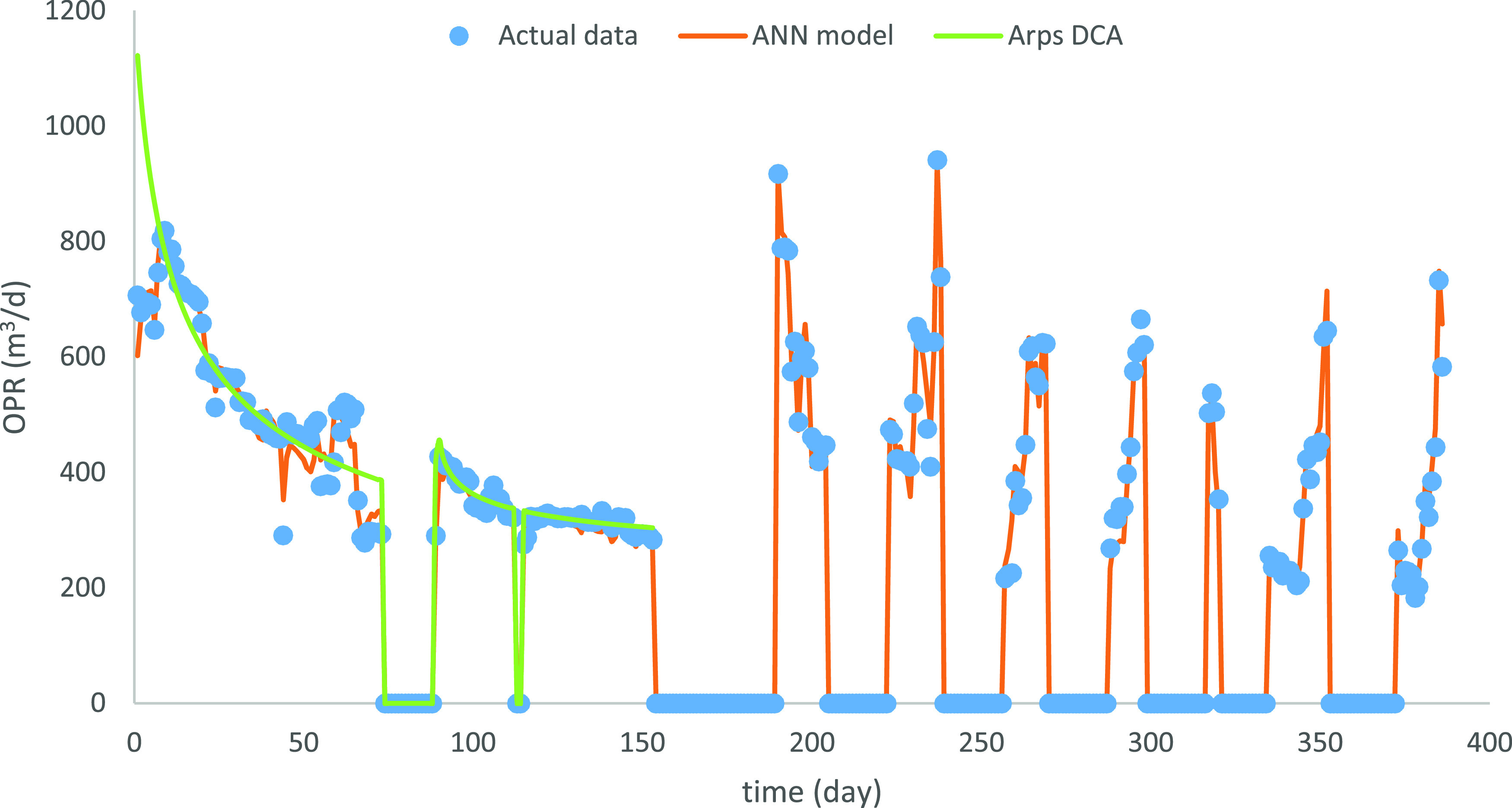
Optimal prediction for OPR.

**Figure 11:**
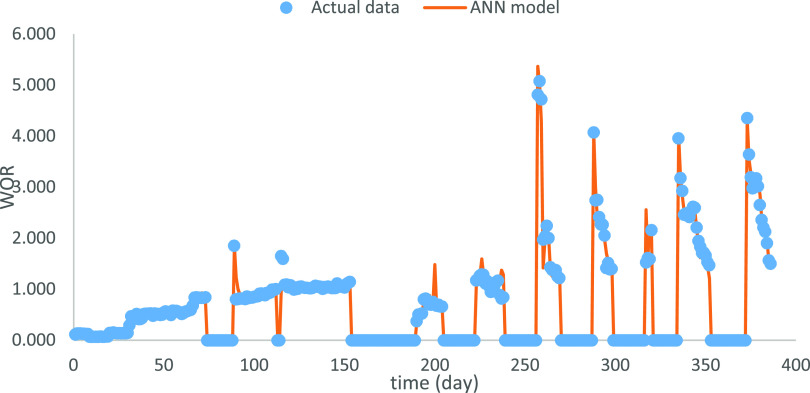
Optimal prediction for WOR.

**Figure 12:**
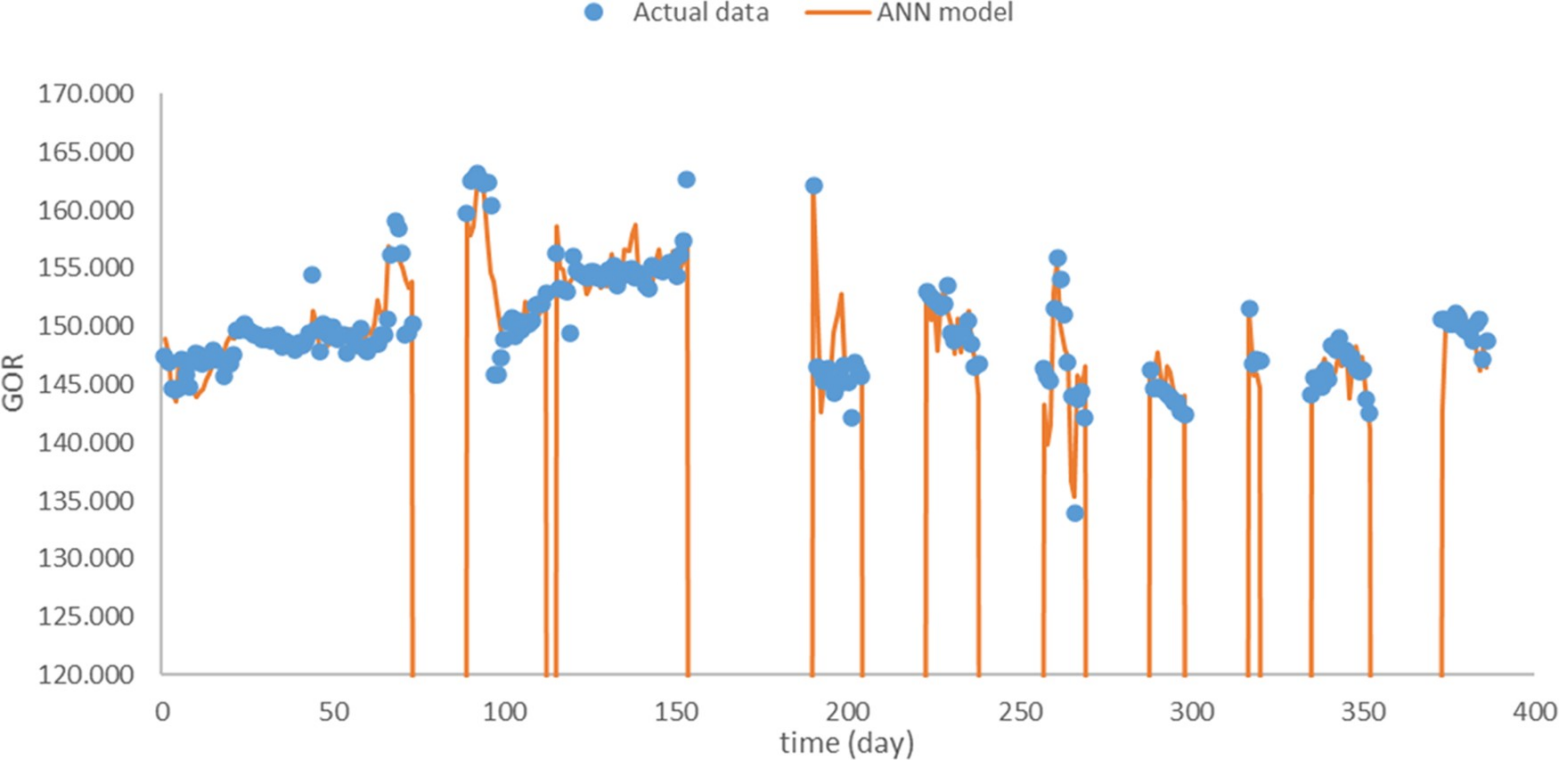
Optimal prediction for GOR.

According to the suitable hyperparameter settings from previous sections, optimal ANN models for OPR, WOR, and GOR were used to estimate the outputs (Figures 10–12). Note that the shut-in periods were excluded in the production estimation since they are completely manually controlled and unpracticable for the WOR and GOR computation.The relative RMSEs reach 6.8, 18.0, and 1.98% for OPR, WOR, and GOR, respectively. The results denote the high accuracy of the data-driven model. The relatively higher error for WOR forecast may be caused by the low correlation between inputs and WOR or the relatively high range of the WOR.

The production performance varies after the shut-in period. Combining the new OPR and WOR plots (Figures 10 and 11), there are two phenomena in the production performance after the shut-in period. The OPR peaks at the beginning of the reproduction period and then declines, e.g., 190–205 days. In this case, the WOR increased but was consistently lower than the level before the shut-in period. The variation might be caused by the usage of the inflow control device, which can partially choke flow to increase the oil production, while delaying the water production increase. However, the OPR will still naturally decrease. Another one is that the OPR begins at a low level and then increases to the peak after the shut-in period, while WOR shows the inverse trend, e.g., 290–300 days. The water injection starting from the shut-in period could cause the variation combined with the expansion of open choke size. The water injection and larger choke size lead to the OPR growth, and the inflow control device delays the water production increase or even reduces the water production, which leads to the decrease of WOR. Well performance could be further analyzed when more information is available.

### 3.6 Discussion

In the previous section (Section 3.5), ANN models with optimal hyperparameter settings were developed and estimated the target outputs (OPR, WOR, and GOR). This section explicitly discussed the strengths and limitations of ANN models.

#### 3.6.1 Comparison with Empirical Model

To verify the effectiveness of the optimal ANN model, the Arps DCA was applied to the OPR estimation. The initial two phases of the production period, production period before and after the first shut-in period, were adopted for the DCA fitting, since only these two phases complied with the production decline trend. The OPR estimation through ANN and DCA is shown in Figure 10, and the relative RMSE of two approaches is shown in Table 8.

**Table 8:** Relative RMSE of OPR Estimation

OPR estimation	ANN (%)	Arps DCA (%)
phase 1	5.85	18.75
phase 2	4.04	7.30

According to the relative RMSE, the ANN model performs better than Arps DCA in both phases. In addition, the relative RMSE in phase 2 is lower than that in phase 1 for both methods, which proves that the estimation methods perform better for the production performance without frequent operation variation. Furthermore, the comparison of relative RMSE decrease, from phase 1 to phase 2 between ANN and Arps DCA, verifies that Arps DCA is more susceptible to the operation condition change. However, Arps DCA requires only production data for the estimation, while the ANN model requires various operation parameters, which could influence its estimation accuracy. Since the accuracy between Arps DCA (7.30% error) and ANN (4.04% error) is close, DCA is suitable for the production estimation when the operation condition is stable, and there is limited operation information. When there is abundant information about operation parameters and/or reservoir parameters, e.g., simulation data or field data, the ANN model could show its strength.

#### 3.6.2 Input Effect

**Figure 13:**
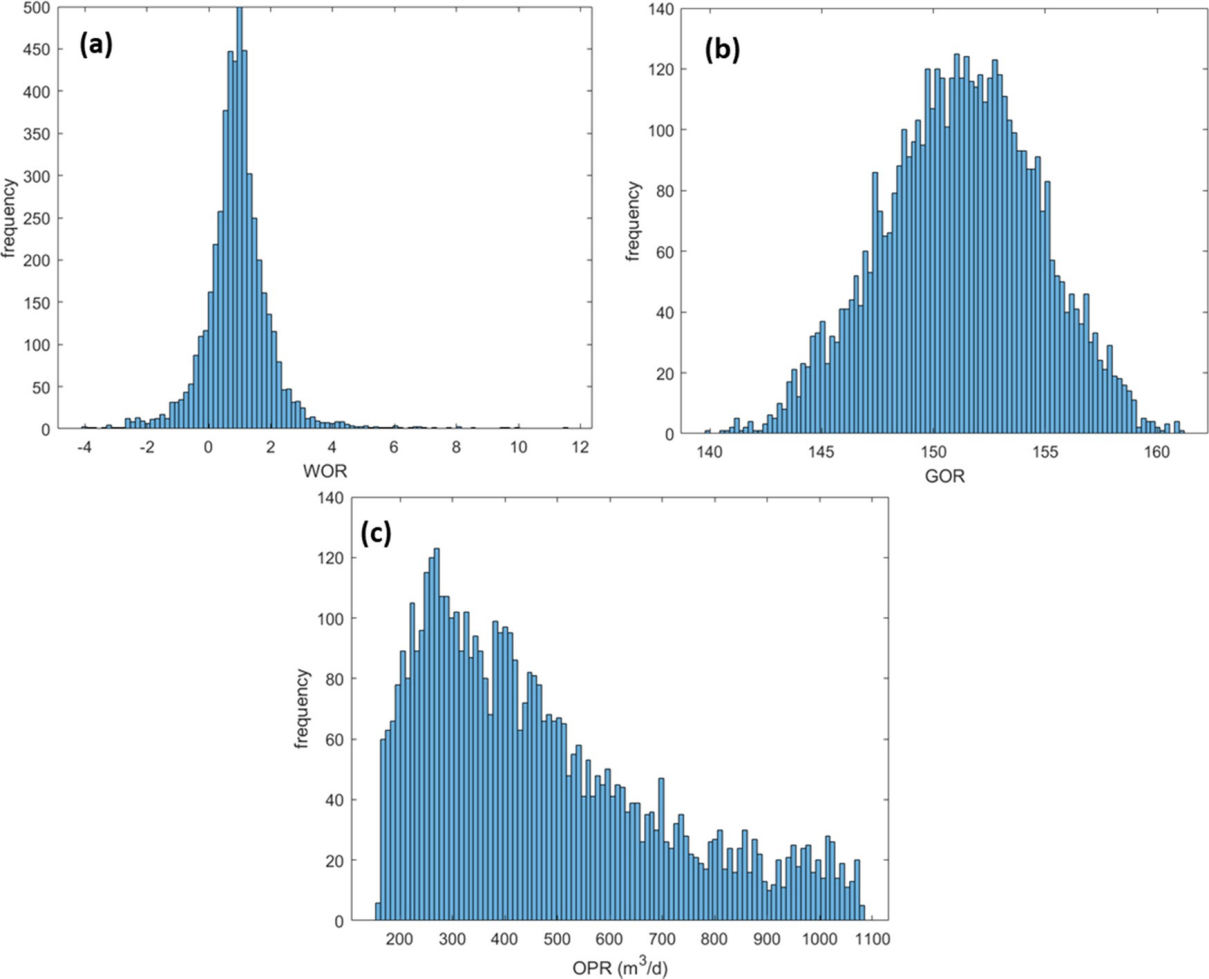
Distribution histogram of outputs. (a) WOR; (b) GOR; and (c) OPR.

ANN models are applied to deal with the real-world problems due to flexibility and efficiency. For the regression problems, e.g., production parameter estimation, ANN models can be regarded as complex functions. Thus, outputs of the probabilistic distribution through Monte Carlo simulation can be applied to ANN models as well. In this section, the input effect for the estimation of OPR, WOR, and GOR were explored through Monte Carlo simulation. First, the input cumulative distribution functions were created based on the input distribution fitting. Then, random numbers were generated and converted to the random input variables through cumulative distribution functions. Finally, the outputs (OPR, WOR, and GOR) were obtained through ANN models. In this study, we ran 5000 simulations to reduce the uncertainty, and the histograms of OPR, WOR, and GOR are shown in Figure 13.

The frequency of the outputs could be derived from Figure 13, which might be helpful for the field development. The GOR ranges from 140 to 160, and the most likely value is around 153, which verified the major status of the reservoir gas. The OPR ranges from 200 to 1000 m^3^/d, and the most likely value is around 280 m^3^/d, which could be applied to oil recovery estimation and economic evaluation. However, the negative value of WOR range is different from the common sense. The possible reason is that the ANN model is the data-driven model, and the low accuracy for WOR estimation led to the probability of outliers, which is the limitation of the ANN model. To solve this issue, physical constraint should be added, e.g., WOR should be higher than 0. The application of probabilistic distribution through the ANN model can be applied in many areas of oil and gas industry, e.g., multiscale analysis of subsurface characterization. However, the physical properties should be notified when applying this approach to avoid unreasonable results.

### 3.7 Future Direction

Some further research can be conducted in the future. For example, the hidden layer number and neuron numbers in hidden layers can be optimized. In addition, the relationship between the physical meaning of inputs and outputs could be considered during the hyperparameter setting, e.g., the pressure difference with the OPR. Furthermore, the possibility of finding a standard optimal ANN model applied to the different periods of the well or two adjacent production wells is still an open question. In addition, for the ANN application in other types of wells or reservoir behavior, e.g., gas coning cases, the optimal hyperparameter setting and effectiveness can be further discussed when the data is available.

## 4 Conclusions

Well production performance is the critical parameter in the economic evaluation in the oil and gas field. This study applied the artificial neural network to estimate the single-well OPR, WOR, and GOR through historical data matches. Different types of neural networks, training, and transfer functions were analyzed to find the optimal setting. The evaluation parameter called relative root mean square error was adopted and combined with statistical parameters to figure out the optimal hyperparameter setting for ANN models stochastically. The results showed the effectiveness of the optimal ANN models in OPR, WOR, and GOR. In addition, the comparison with DCA and the inputs effect illustrate the strength and limitation of ANN models. More research directions could be deeply explored based on this study, e.g., the transfer learning application in production estimation.

## Appendix A

The appendix lists the ANN hyperparameters for Section 3.3, including the neural network, training, and transfer function settings for OPR, WOR, and GOR. There are 45 ANN groups for each output, and the numbers within Table 9–11 deno te the ANN group numbers. Table 9–11 also show the hyperparameter abbreviations.

**Table 9:** Network Types for Hyperparameter Groups of OPR, WOR, and GOR

neural network	production rate	WOR	GOR
function fitting (fitnet)	1–15	1–15	1–15
feedforward (newff)	16–30	16–30	16–30
cascade-forward (newcf)	31–45	31–45	31–45

**Table 10:** Training Functions for Hyperparameter Groups of OPR, WOR, and GOR

training function (backpropagation)	production rate	WOR	GOR
Levenberg–Marquardt (trainlm)	1, 16, 31, 23–30,	1, 16, 31, 23–30,	1, 8–16, 23–31, 38–45
Bayesian regularization (trainbr)	2, 17, 32, 8–15, 38–45	2, 17, 32,	2, 17, 32,
resilient backpropagation (trainrp)	3, 18, 33	3, 18, 33, 8–15, 38–45	3, 18, 33
BFGS quasi-Newton (trainbfg)	4, 19, 34	4, 19, 34	4, 19, 34
conjugate gradient with Fletcher–Reeves updates (traincgf)	5, 20, 35	5, 20, 35	5, 20, 35
conjugate gradient with Powell–Beale restarts (traincgb)	6, 21, 36	6, 21, 36	6, 21, 36
conjugate gradient with Polak–Ribiére updates (traincgp)	7, 22, 37	7, 22, 37	7, 22, 37

**Table 11:** Transfer Functions for Hyperparameter Groups of OPR, WOR, and GOR

transfer function	production rate	WOR	GOR
soft max (softmax)	1–7, 16–22, 31–37	1–7, 16–22, 31–37	1–7, 16–22, 31–37
log-sigmoid (logsig)	8, 23, 38	8, 23, 38	8, 23, 38
triangular basis (tribas)	9, 24, 39	9, 24, 39	9, 24, 39
saturating linear (satlin)	10, 25, 40	10, 25, 40	10, 25, 40
symmetric saturating linear (satlins)	11, 26, 41	11, 26, 41	11, 26, 41
positive linear (poslin)	12, 27, 42	12, 27, 42	12, 27, 42
linear (purelin)	13, 28, 43	13, 28, 43	13, 28, 43
radial basis (radbas)	14, 29, 44	14, 29, 44	14, 29, 44
normalized radial basis (radbasn)	15, 30, 45	15, 30, 45	15, 30, 45
